# Practitioners' Perceptions of Augmented and Virtual Reality in Endodontic Treatment Planning: A Pilot Study

**DOI:** 10.1111/iej.70143

**Published:** 2026-03-15

**Authors:** Marcel Reymus, Nils Werner, Falk Schwendicke, Ralf Krug

**Affiliations:** ^1^ Department of Conservative Dentistry, Periodontology and Digital Dentistry LMU University Hospital, LMU Munich Munich Germany; ^2^ Department of Conservative Dentistry and Periodontology University Hospital Würzburg Würzburg Germany

## Abstract

**Aim:**

To develop a streamlined software workflow for converting cone‐beam computed tomography (CBCT) data into augmented reality (AR) and virtual reality (VR) formats, and to evaluate dental practitioners' perceptions of these advanced visualisation modalities compared to conventional CBCT analysis for endodontic treatment planning.

**Methodology:**

A custom add‐on for Blender (an open‐source 3D modelling software), named VirtualEndo, was developed to automate the processing of AI‐segmented CBCT scans into AR‐ and VR‐compatible formats. Thirty dentists evaluated two diagnostically challenging cases using four methods: conventional CBCT analysis, AI‐driven 3D segmentation, AR visualisation on iPad and VR visualisation with Meta Quest 3. The presentation order was randomised, and participants completed a standardised training protocol. Perceptions regarding diagnostic confidence, usability, information extraction, and clinical relevance were assessed using 4‐point Likert scales. Statistical analysis employed Friedman tests with post hoc Wilcoxon signed‐rank tests and Bonferroni correction.

**Results:**

Significant differences were found across all evaluated categories (*p* < 0.05). Conventional CBCT was rated significantly inferior to Segmentation, VR, and AR for information extraction (canal course: *W* = 0.68; canal number: *W* = 0.56) and usability (*W* = 0.40). No significant differences existed between the three advanced visualisation methods. Segmentation was most frequently selected as clinically most relevant (40%), followed by VR (20%). For canal detection, differences were small (*W* = 0.10) with no method demonstrating clear superiority.

**Conclusions:**

Modern 3D visualisation technologies were perceived as significantly superior to conventional 2D CBCT slice analysis, primarily by presenting pre‐integrated anatomical models that reduce the perceived cognitive burden of mental 3D reconstruction from 2D slices. Screen‐based segmentation was favoured for pragmatic workflow integration, though immersive technologies showed promise if adoption barriers are addressed.

## 1 Introduction

A comprehensive endodontic diagnosis and treatment plan is essential for successful root canal therapy. The process has been significantly enhanced through the shift from two‐dimensional (2D) to three‐dimensional (3D) imaging. For decades, diagnosis and treatment planning were guided by 2D radiographs, which, despite their utility, are constrained by inherent limitations such as geometric distortion and the superimposition of anatomical structures (Gliga et al. 2023; Huumonen and Ørstavik 2002; Sarsam et al. 2025). Two‐dimensional radiographic imaging is fundamentally limited by overlapping structures, geometric distortion, and anatomical noise, preventing accurate visualisation of 3D structures essential for endodontic diagnosis (AlMohareb et al. 2022). Lo Giudice et al. demonstrated that conventional radiography presents critical limitations including 3D anatomic compression, geometric alterations of up to 14% in orthopantomography, and anatomical obstacles that can obscure regions of interest, leading to diagnostic omissions of periradicular pathologies (Lo Giudice et al. 2018). This ‘anatomical noise’ can obscure critical details of complex root canal systems, forcing clinicians to rely on subjective mental reconstructions of 3D anatomy. The advent of cone‐beam computed tomography (CBCT) marked a paradigm shift, providing an anatomically accurate volumetric dataset of high resolution that allows for the non‐invasive exploration of maxillofacial structures, free from the distortions of planar imaging. This has unequivocally elevated the standard of care for complex endodontic cases involving challenging anatomy, resorptive defects, or proximity to vital structures (Patel et al. 2014, 2019). CBCT's multiplanar evaluation eliminates superimposition and anatomical noise, providing superior diagnostic capabilities particularly for complex root canal anatomy and periapical lesion detection (Mashyakhy et al. 2021). CBCT represents thus a valuable diagnostic and treatment planning tool in endodontic practice, though its appropriate clinical application requires understanding of scenarios where it provides diagnostic advantage over conventional radiography (Chan et al. 2023). CBCT has revolutionised the assessment of root and root canal morphology by overcoming limitations of conventional 2D radiographs including distortions, superimpositions, and inability to visualise three‐dimensional complexity (Karobari et al. 2023). However, while CBCT provides 3D data, the primary mode of interaction has remained confined to a 2D interface—evaluating a series of 2D planes on the computer monitor. This imposes a significant perceived cognitive burden: the practitioner must mentally integrate multiple axial, coronal, and sagittal slices into a coherent 3D model of the clinical situation. Such a process is not only time‐consuming and mentally taxing but also inherently prone to misinterpretation, especially in anatomically challenging cases. Flattening‐based visualisation techniques aim to reduce cognitive complexity by projecting 3D anatomical structures into simplified 2D representations, though this approach still requires significant mental integration from clinicians (Kreiser et al. 2018). This cognitive gap between 3D data acquisition and 2D visualisation represents a current bottleneck in the diagnostic workflow and underscores the need for more intuitive visualisation and interaction technologies. Recent innovations have made Augmented Reality (AR) and Virtual Reality (VR) the next significant step in digital evolution, allowing for the liberation of 3D datasets from the confines of traditional monitors. These immersive technologies serve as advanced visualisation layers that allow clinicians to interact with patient‐specific anatomy in a true‐to‐scale, fully immersive 3D environment.

Dynamic 3D visualisations support anatomical learning by providing continuous viewpoints and reducing the cognitive demand of mentally reconstructing three‐dimensional structures from static two‐dimensional images, though individual spatial abilities significantly influence learning outcomes (Berney et al. 2015). The potential of these technologies in the field of endodontic education to enhance students' spatial understanding of the anatomy has been reported in several studies (Alsalleeh et al. 2024; Diegritz et al. 2024; Ba‐Hattab et al. 2023). A systematic review of VR/AR in dental education confirmed motor skill acquisition comparable to traditional methods, with primary applications in undergraduate training for restorative dentistry, oral surgery, and endodontics, though standardisation and accreditation remain ongoing challenges (Dzyuba et al. 2025). Recent systematic reviews demonstrate evidence that virtual reality is as effective as traditional learning techniques in endodontic education, enhancing skill proficiency, reducing procedural errors, and improving anatomical comprehension among undergraduate dental students (Deshpande et al. 2025). Saghiri et al. (Saghiri et al. 2022) even stated that virtual reality dental teaching environments integrating 3D information achieve significantly better educational results compared to 2D teaching materials, with augmented reality additionally enabling 360° spatial visualisation that effectively overcomes limitations of conventional teaching approaches. Despite its obvious potential, the technologies' widespread clinical adoption has been hindered by numerous significant barriers. Challenges that include the high initial capital investment for hardware and software, a steep learning curve for the associated digital workflows, and difficulties in seamlessly integrating these time‐consuming processes into the efficiency‐driven environment of a busy clinical practice have been discussed (Fahim et al. 2022; Bencharit et al. 2025). There is risk of a potential mismatch between the ‘technology push’ and the ‘clinician pull’ from the end‐user community. The barriers to entry imply that the perceived benefits may not yet outweigh the practical and financial costs for many practitioners, indicating a possible disconnect between the problems the technology aims to solve and the most pressing needs of clinicians. This deficit is acknowledged, calling for studies that move beyond procedural metrics to evaluate factors such as clinician confidence, cost‐effectiveness, and the practical barriers to implementation. The ultimate success or failure of AR and VR in endodontics will not be determined by technical specifications, but by their clinical viability. Therefore, understanding the human factors—usability, perceived value, and the willingness of practitioners to overcome adoption barriers—has become the most critical and underexplored area of inquiry that will shape the future of these technologies in clinical practice.

Building on these points, this study was designed with two main objectives. The first was to address the current bottleneck in the clinical workflow; the time‐consuming and technically demanding process of transforming CBCT data into formats suitable for AR/VR visualisation. While Artificial Intelligence (AI) has made segmentation of CBCT scans faster and more user‐friendly. Novel AI‐powered tools utilising 3D U‐Net architecture demonstrate highly accurate and time‐efficient automated root canal segmentation on CBCT (DSC: 89%–93%, time reduction: 54‐fold), surpassing manual segmentation performance and significantly reducing workflow bottlenecks (Santos‐Junior et al. 2025). Artificial intelligence significantly enhances endodontic practice by automating segmentationof fine anatomical structures such as pulp chambers and root canals, though clinical validation with in vivo datasets remains essential for reliable clinical implementation (Fontenele and Jacobs 2025). The resulting datasets—most often exported as Standard Tessellation Language (STL) files—still require several additional and technically sensitive steps before they can be rendered in immersive environments. This fragmented workflow not only limits efficiency but also reinforces the very perceived cognitive burden that AR and VR are intended to reduce. To overcome this limitation, a dedicated software extension was developed that automates this process. The second objective was to explore how dental practitioners perceive the added value of AR and VR in endodontic treatment planning. This study sought to evaluate practitioners' perspectives regarding the utility, benefits, and barriers of AR/VR integration. Particular attention was given to aspects such as improved spatial understanding, diagnostic confidence and concerns about usability.

## 2 Materials and Methods

This pilot study was reviewed and approved by the Ethics Committee of the Medical Faculty of the University Munich (ID: 25‐0101), reviewed and approved this pilot study. The was carried out strictly in accordance with the principles of good clinical practice and the Declaration of Helsinki. Furthermore, this manuscript follows the guidelines for reporting observational studies (STROBE).

### 2.1 Software Development

A custom Blender (blender.org, Blender Foundation, Amsterdam, Netherlands) add‐on called *VirtualEndo* was developed specifically for the workflow to automate the processing and conversion of STL files derived from AI driven segmented CBCT scan. The add‐on was programmed in Python 3 using Blender's API (Application Programming Interface). The add‐on implements an intelligent file categorization algorithm that automatically identifies and sorts STL files based on standardised naming conventions:

- Pulp files: recognised by the pattern pulp_XX.stl (e.g., pulp_11.stl for the maxillary right central incisor, where *XX* = FDI tooth number).
- Tooth files: identified by the pattern tooth_XX.stl.
- Jaw bone files: detected as mandible.stl and maxilla.stl.

This automated recognition system eliminates manual file sorting and reduces the potential for human error during batch processing. The add‐on automatically assigns anatomically appropriate materials to each imported object category using Blender's shader system (surface colour, transparency and material properties). These properties were calibrated to provide optimal visual distinction between anatomical structures while maintaining realistic appearance for educational and presentation purposes. The add‐on performs the following automated processing steps:

1. Scene Preparation: Clears the existing Blender scene to ensure clean processing.
2. STL Import: Sequentially imports all recognised STL files using Blender's built‐in STL import functionality.
3. Material Application: Assigns category‐appropriate materials based on file classification.
4. Geometric Processing:
  - Applies uniform to convert from STL units to Blender units.
  - Centers objects at world origin for consistent positioning.
  - Applies smooth shading to improve visual quality.
5. Export Preparation: Selects all imported objects for subsequent export operations.

The add‐on supports three distinct export formats, each optimised for specific use cases: (1) USDZ Export: to generate iOS‐compatible AR files. (2) GLB Export: for universal 3D format compatibility. (3) FBX Export: for native compatibility with major VR development engines (Unity 3D, Unreal Engine).

To promote reproducibility and facilitate further development within the research community, the complete add‐on was made publicly available through a GitHub repository (https://github.com/mreymus/VirtualeEndo‐Converter). The project was released under the MIT Licence, which permits unrestricted use, modification, and distribution of the software for both academic and commercial purposes. The repository includes comprehensive documentation and installation instructions to facilitate adoption by other researchers. This open‐source approach aligns with principles of open science and ensures long‐term availability of the research tools, independent of commercial software licensing constraints.

**FIGURE 1:**
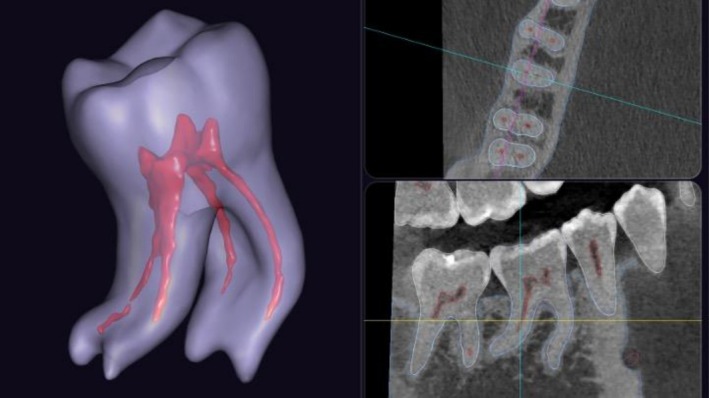
Screenshots of the investigated mandibular molar in segmented view by Diagnocat (left) and corresponding annotated CBCT slices (right).

**FIGURE 2:**
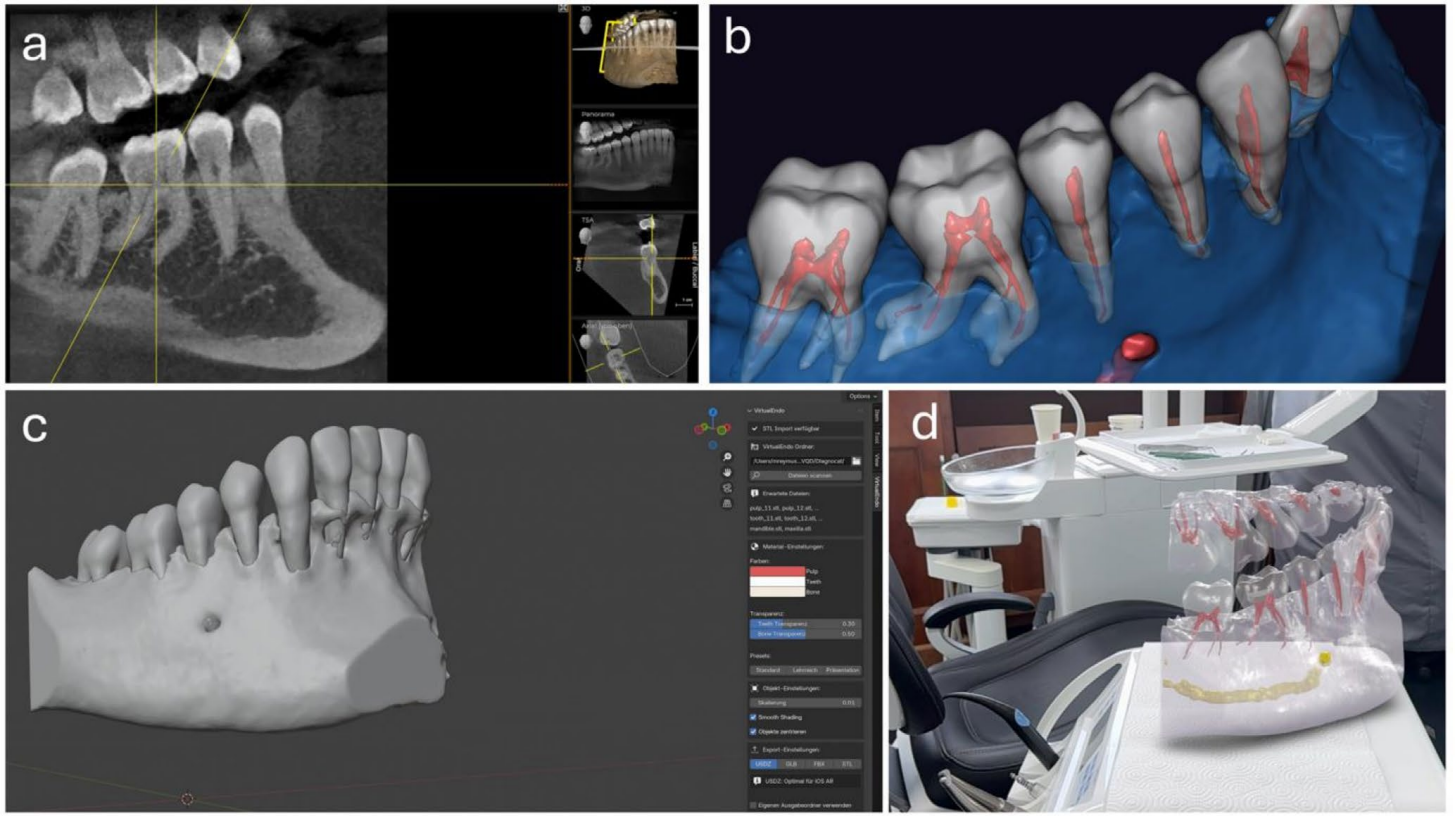
Workflow from the CBCT scan (a) to the segmentation via Diagnocat (b) to Conversion via *VirtualEndo* (c) to displaying the situation in Augmented Reality (d).

Image Preparation: For this study, two clinical cases were randomly selected to represent common yet diagnostically challenging anatomical variations in endodontics. The first case was a mandibular molar exhibiting a complex bifurcation of the canal system within the distal root (Figure 1). The second was a maxillary molar with a second mesiobuccal canal (MB2) that was confluent with the primary mesiobuccal canal (MB1). For both cases, anonymised, high‐resolution CBCT scans with a small field of view (FOV) were available (Orthophos SL 3D, Dentsply Sirona, Bensheim, Germany; 5 × 5 cm FOV, HD mode, 85 μm voxel size). The CBCT datasets were first processed using an AI‐driven segmentation software (Diagnocat, Diagnocat Ltd., London, UK) to generate high‐fidelity 3D models of the relevant anatomical structures, which were exported as STL files. These files were then processed with the custom *VirtualEndo* Blender add‐on, as previously described, to create optimised models for each of the three‐dimensional visualisation platforms used in the study (Figure 2).

### 2.2 Clinical Suitability

Data collection was conducted between March 2025 and August 2025 at the Department of Conservative Dentistry of the University Hospital of Würzburg, Germany and at a private endodontic continuing‐education program (Tec2, tec2‐endo.de, Memmingen, Germany). Eligible participants were licensed dentists with specialisation in endodontics and a minimum of 2 years in CBCT experience for endodontic diagnosis. Their experience in utilising CBCT for diagnostic purposes ranged from 2 to 15 years, with a mean of 6.7 years (SD = 3.3). This variability in CBCT experience was not incorporated as a covariate given the exploratory nature and sample size of this pilot study, but represents a potential source of residual variability. Thirty dentists were approached and agreed to participate voluntarily, completing the full study protocol (100% participation rate, no missing data) (18 male, 12 female, mean age of 39.4 years, SD = 7.1). The sample size of 30 participants was based on feasibility for this pilot study and is consistent with similar perception studies. The cohort's professional experience ranged from 3 to 29 years, with a mean of 13.1 years (SD = 7.0). Their experience in utilising CBCT for diagnostic purposes ranged from 2 to 15 years, with a mean of 6.7 years (SD = 3.3). Each participant was tasked with formulating a hypothetical endodontic treatment plan (e.g., number of canals to be treated, curvatures of canals to be expected, etc.) for both clinical cases. These plans served to ensure meaningful clinical engagement with each visualisation modality; systematic comparison of treatment plan content across modalities, however, was not evaluted. The evaluation process for each case was structured in two stages. First, participants performed a conventional diagnostic evaluation of the CBCT datasets using OsiriX Lite (v.12.5, Pixmeo SARL, Switzerland) on a 16‐in. MacBook Pro (Apple Inc., Cupertino, CA, USA). The evaluation utilised standard multiplanar reformation (MPR) with synchronised axial, coronal, and sagittal views, representing the predominant clinical workflow for endodontic CBCT interpretation. Advanced features such as curved planar reformatting, volume rendering, or maximum intensity projections were not employed, as these would have required additional training. Subsequently, the participants reviewed the same case using three advanced 3D visualisation modalities:

1. Segmented Model on Diagnocat.com: An interactive 3D model on the MacBook Pro screen (MacBook Pro 16‐in.) (Apple Inc.; Liquid Retina XDR display, 3456 × 2234 pixels), which could be freely rotated, translated, and zoomed. The transparency of individual structures (e.g., tooth, pulp, jawbone) could be adjusted by the user.
2. Augmented Reality (AR): An AR visualisation projected onto the real‐world environment via an 11‐in. iPad Pro (Apple Inc.; iquid Retina XDR display, 2388 × 1668 pixels, ProMotion 120 Hz).
3. Virtual Reality (VR): A fully immersive VR experience using a Meta Quest 3 headset (Meta Platforms Inc., Menlo Park, CA, USA; display resolution: 2064 × 2208 pixels per eye, refresh rate: 120 Hz, field of view: 110°), allowing for an in‐depth, true‐to‐scale exploration of the anatomy.

To mitigate potential order effects, the sequence of the three advanced visualisation modalities (Segmentation, AR, and VR) was individually randomised for every participant. A computer‐generated randomization sequence was created using a custom Python script, ensuring that each of the six possible presentation orders was assigned to five participants, achieving complete counterbalancing across the study cohort. To control for variations in technical proficiency, a standardised onboarding protocol was implemented, as none of the participants reported prior experience with the tested visualisation modalities. The training for each modality involved two stages: first, a live demonstration of all features by a designated study investigator. Second, participants navigated a separate 3D test case (not used in the main study) by themselves. This hands‐on session allowed participants to ask questions and familiarise themselves with the controls until they expressed confidence in their ability to perform the diagnostic evaluation. Following the evaluation using both the standard CBCT view and the three 3D modalities, participants were asked to complete a survey. The questionnaire was designed to assess several key aspects on a 4‐point Likert Scale (from 1 = ‘applies’ to 4 = ‘does not apply’). The survey focused on:

- Information Extraction: How effectively they could identify the precise number and spatial course of the root canals using each method.
- Perceived Usability: The ease of use and intuitiveness of interacting with each visualisation tool.
- Influence of Usability: The extent to which the usability of the technology impacted their ability to extract clinically relevant anatomical information.

Clinical relevance was assessed using a single‐choice categorical question asking participants to identify which method they considered most relevant for clinical use, with five response options: CBCT, Segmentation, VR, AR, or ‘all equally important.’

The complete survey questionnaire is detailed in Supporting Information 1.

### 2.3 Statistical Analysis

For the inferential statistical analysis, non‐parametric tests were employed due to the ordinal nature of the rating scales and the dependent sample design (repeated measures). Given the repeated‐measures design with two cases per rater, the hierarchical data structure was considered. Ratings were aggregated across the two cases for each participant prior to analysis, treating each participant's mean rating as the unit of analysis. The Friedman test inherently accounts for within‐subject dependencies in repeated measures. While cumulative link mixed models (CLMMs) for ordinal data could provide additional insights by modelling case‐level and rater‐level random effects, our sample size of 30 participants limits the reliable estimation of variance components. The Friedman test was first conducted for each evaluated category to determine if any global statistically significant differences existed among the four methods. To quantify the magnitude of any observed differences, the corresponding effect size was calculated using Kendall's *W*. If the Friedman test yielded a significant result (*p* < 0.05), post hoc pairwise comparisons were performed using the Wilcoxon signed‐rank test to identify which specific methods differed from one another. To counteract the problem of multiple comparisons, a Bonferroni correction was applied to the significance level. For the six pairwise comparisons in each category, the adjusted significance level (α) was set to *p* < 0.0083. All statistical analyses were conducted in the R environment for statistical computing (Version 4.2.3; R Foundation for Statistical Computing, Vienna, AT) on a macOS platform (Apple Inc.). The hypothesis was that there are no differences between the different visualisation methods.

## 3 Results

30 participating dentists were analysed to identify statistically significant differences between the four evaluated methods: CBCT, Segmentation, VR, and AR. When asked to identify the single most clinically relevant method, Segmentation was the clear preference, chosen by 40.0% (*n* = 12) of the participants. VR was selected by 20.0% (*n* = 6). The conventional CBCT analysis, AR, and the option ‘all of them are equally important’ each received 13.3% (*n* = 4) of the votes. The comparative analysis revealed statistically significant differences across all evaluated categories, with the magnitude of these differences ranging from small to large effects. A consistent pattern emerged where the conventional CBCT method was frequently rated as significantly inferior to the more novel methods of Segmentation, VR, and AR. In contrast, no statistically significant differences were found when comparing these three novel methods against each other in any category. The overall results of the comparisons are presented in Table 1.

**TABLE 1:** Results of each comparison between different methods.

Category and comparison	*p*	Kendall's *W* (Effect size)
Usability (global test)	**< 0.0001**	0.40 (Medium)
CBCT versus Segmentation	**< 0.0001**	
CBCT versus VR	**0.00059**	
CBCT versus AR	**< 0.0001**	
Segmentation versus VR	0.356	
Segmentation versus AR	1.000	
VR versus AR	0.248	
Canal detection (global test)	**< 0.0001**	0.10 (Small)
CBCT versus Segmentation	0.027	
Fried	**0.0003**	
CBCT versus AR	0.07	
Segmentation versus VR	0.328	
Segmentation versus AR	0.634	
VR versus AR	0.288	
Info extraction (canals) (global test)	**< 0.0001**	0.56 (Large)
CBCT versus Segmentation	**< 0.0001**	
CBCT versus VR	**< 0.0001**	
CBCT versus AR	**< 0.0001**	
Segmentation versus VR	0.484	
Segmentation versus AR	0.072	
VR versus AR	0.346	
Info extraction (course) (global test)	**< 0.0001**	0.68 (Large)
CBCT versus Segmentation	**< 0.0001**	
CBCT versus VR	**< 0.0001**	
CBCT versus AR	**< 0.0001**	
Segmentation versus VR	1.000	
Segmentation versus AR	1.000	
VR versus AR	1.000	

*Note:* Results of the Friedman test and post hoc Wilcoxon signed‐rank tests. Effect size is reported as Kendall's *W*. Significant results are bold (*p* < 0.0083).

Abbreviations: AR, augmented reality; CBCT, cone‐beam computed tomography; VR, virtual reality.

**FIGURE 3:**
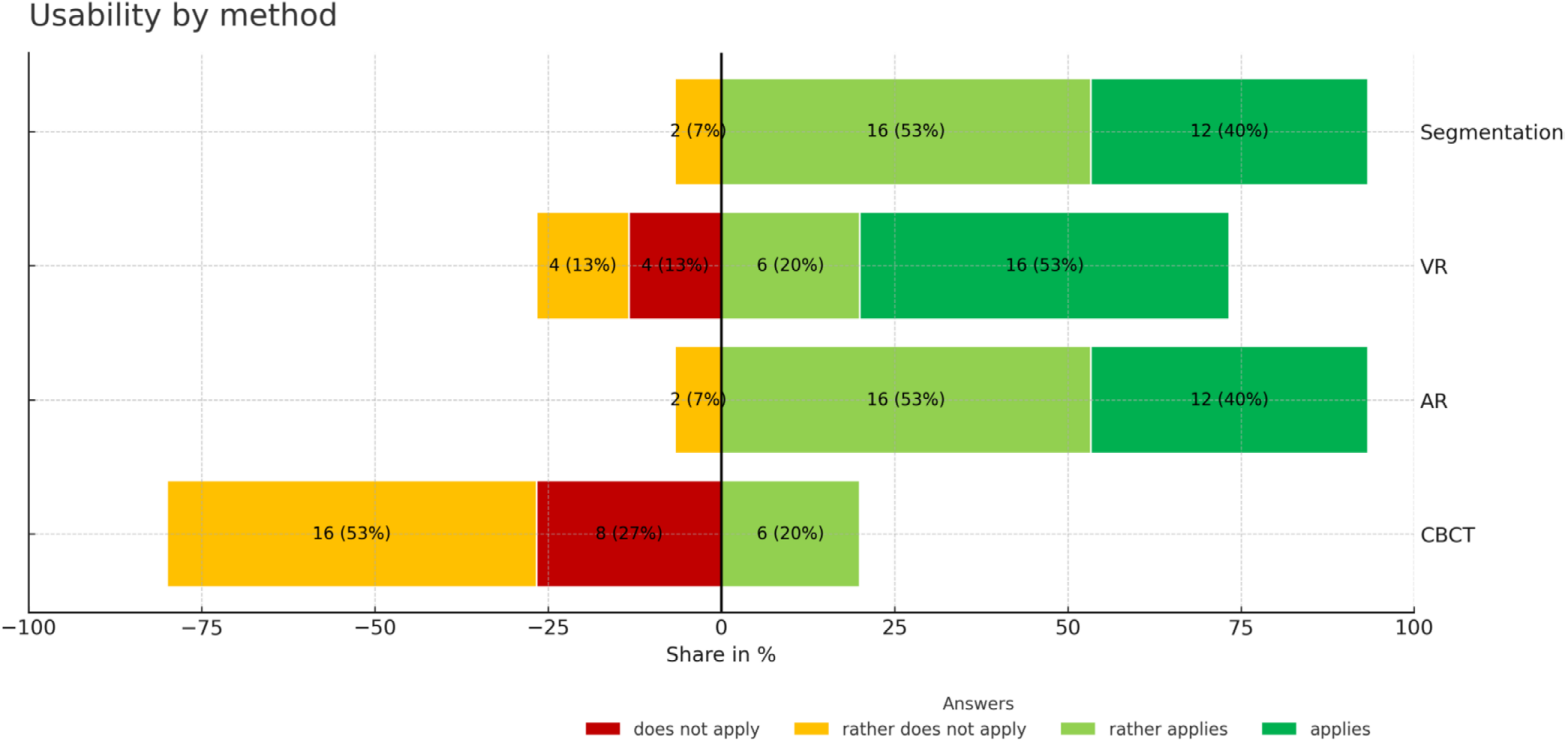
Usability ratings of the different visualisation methods for endodontic treatment planning.

**FIGURE 4:**
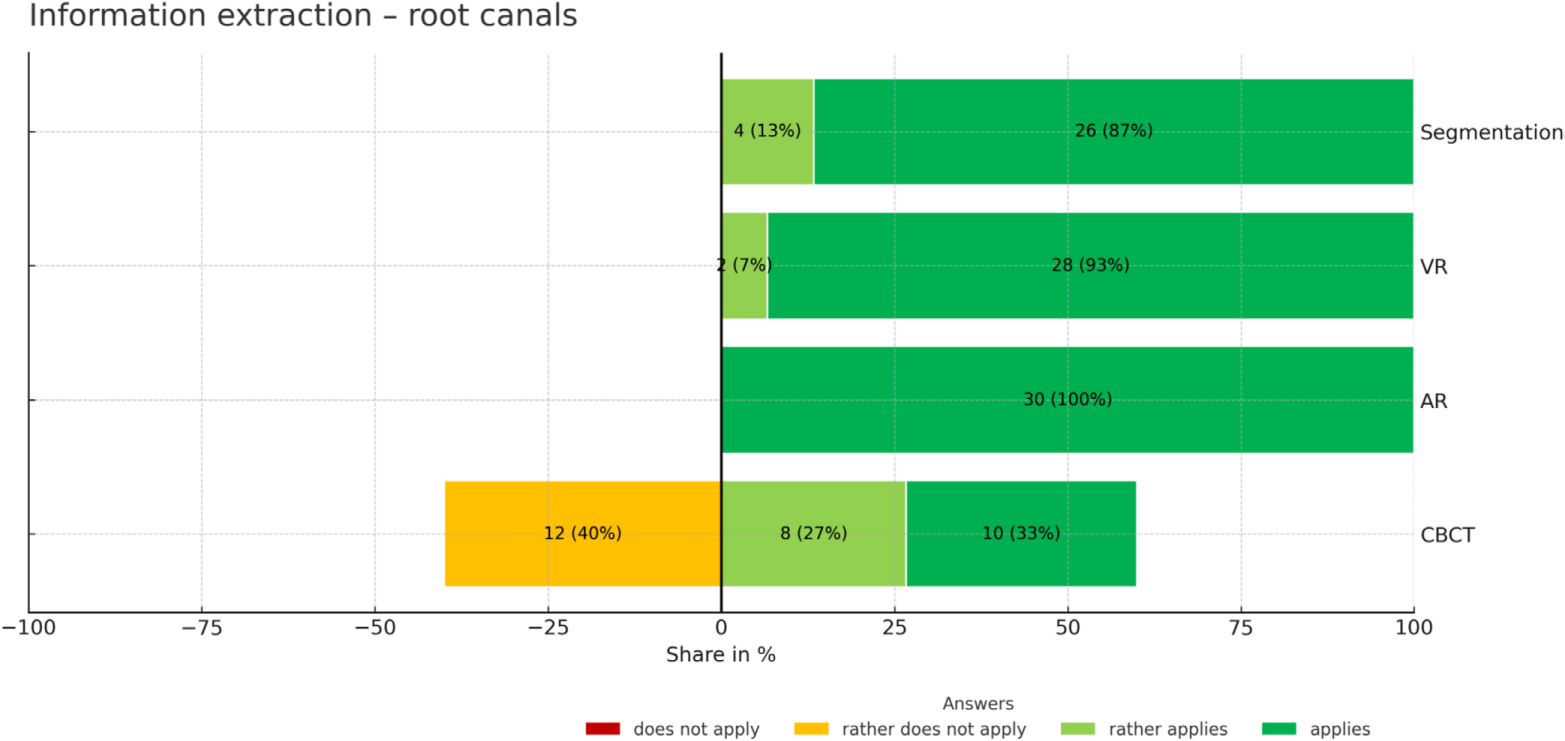
Ratings for the detection of root canal numbers by visualisation method.

**FIGURE 5:**
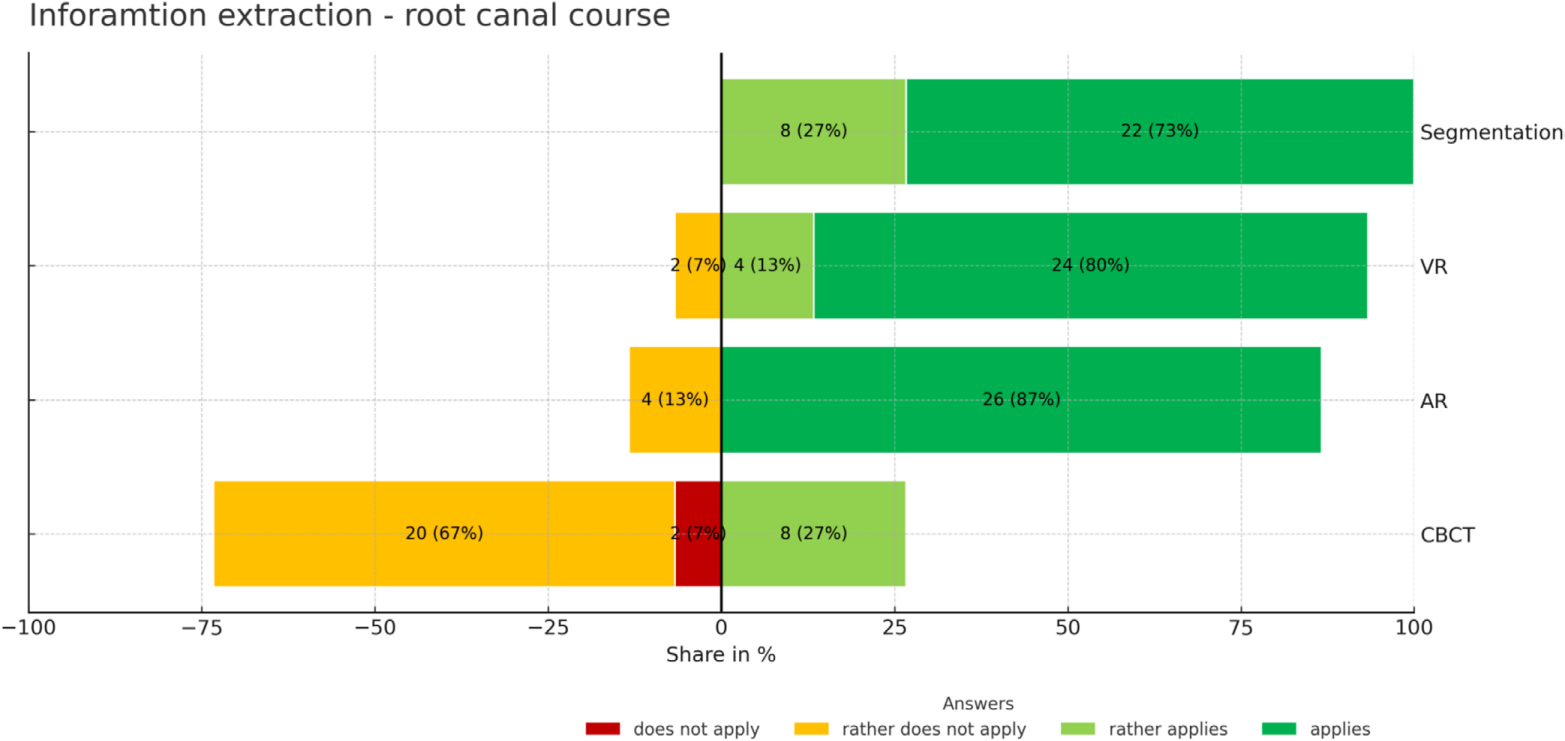
Ratings for the detection of root canal course by visualisation method.

Usability & Information Extraction: The differences among the methods were most pronounced in the categories related to information extraction and usability. The Friedman test was highly significant for Information Extraction (Canal Course) (*χ* ^2^(3) = 61.09, *p* < 0.0001), with a large effect size (*W* = 0.68). A similarly large effect was found for Information Extraction (Canals) (*χ* ^2^(3) = 50.29, *p* < 0.0001, *W* = 0.56). For usability, the test was also significant (*χ* ^2^(3) = 35.85, *p* < 0.0001), corresponding to a medium effect size (*W* = 0.40). For all three categories, subsequent post hoc analysis confirmed that the CBCT method received significantly poorer ratings than Segmentation, VR, and AR (all *p*‐values < 0.0083). The results of the comparisons between the visualisation methods are presented in Figures 3, 4, 5.

Despite the significant global finding, the post hoc pairwise comparisons failed to identify a significant difference between nearly all pairs of methods after applying the stringent Bonferroni correction. However, post hoc analysis revealed a statistically significant difference between CBCT and VR for canal detection (*p* = 0.0003). The results are presented in Table 1.

## 4 Discussion

The transition from 2D radiographic interpretation to immersive 3D visualisation holds immense promise for enhancing endodontic diagnostics, yet its clinical integration has been slow. This study aimed to bridge this gap by developing a streamlined software workflow, *VirtualEndo*, and evaluating clinicians' perceptions of advanced visualisation modalities. Our results reveal a significant preference for modern 3D methods—namely Segmentation, VR, and AR—over conventional CBCT analysis, particularly in terms of usability and the perceived quality of information extraction. This finding suggests that while CBCT provides the essential data, its traditional 2D‐slice interface is a key limiting factor, imposing a perceived cognitive burden that immersive technologies can alleviate.

To ensure experience within the technology of CBCT imaging, all participants were long‐term users. When asked about their primary indications for using CBCT in endodontics, the most frequently reported reason was for the diagnosis and treatment planning of suspected complex root canal anatomy, which was compared in this study in two clinical cases.

A central finding of this study was that clinicians' perceptions clearly distinguished between data acquisition (obtaining a CBCT scan) and data interpretation (the cognitive process of extracting diagnostic information from that data). While CBCT as an imaging modality received no criticism, its conventional interpretation method, namely reviewing sequential 2D slices, was consistently rated as inferior to methods presenting pre‐integrated 3D models. So, the conventional interpretation of CBCT was consistently rated as significantly inferior, a result supported by the large effect sizes for information extraction (*W* = 0.56 and *W* = 0.68). While CBCT itself is the gold standard for evaluating root canal anatomy, our findings indicate that its clinical utility is fundamentally constrained by its nature of interpretation, that is, reviewing several 2D slices on all axes. The high ratings for Segmentation, VR, and AR, which showed no statistical difference among themselves, underscore that clinicians value any method that presents a pre‐integrated 3D model, thereby offloading the task of mental reconstruction.

It is essential to emphasise that a high‐quality, diagnostically robust CBCT scan serves as the prerequisite for any subsequent visualisation. If this initial dataset is of poor quality or contains significant artefacts, any further processing risks obscuring critical structures, potentially leading to a treatment plan based on a distorted or incomplete anatomical representation. Of equal importance is the accuracy of the AI‐driven segmentation. The underlying algorithm must inevitably and faithfully delineate each structure based on the CBCT data, without inferring or extrapolating beyond the provided information. Therefore, the clinician retains the ultimate responsibility to verify the segmentation output against the original CBCT slices to identify any discrepancies. Current segmentation algorithms may struggle with complex pathologies such as external root resorption or dens invaginatus. In such cases, the clinician must meticulously scrutinise the automated segmentation to ensure its clinical fidelity.

Interestingly, while Segmentation was chosen by 40% of participants as the most ‘clinically relevant’ method, its performance in usability and information extraction was statistically indistinguishable from VR and AR. This may reflect a pragmatic compromise by clinicians: screen‐based 3D models (Segmentation) offer a significant upgrade over 2D slices without requiring the adoption of new hardware like VR headsets or AR‐compatible tablets. This points to a crucial barrier to adoption: workflow disruption. Clinicians appear to favour the path of least resistance, preferring solutions that enhance their current setup rather than those that require a paradigm shift in their interaction with patient data. The financial and temporal overhead associated with immersive technologies—from the procurement of a VR headset to the time required to load models for AR viewing—appears to be a significant deterrent. Consequently, the workflow that remains confined to a 2D screen is still perceived as the most time‐ and cost‐effective. However, this perception could evolve if immersive modalities demonstrate unique and compelling advantages over screen‐based 3D analysis. If future developments, potentially through software like the one described, can leverage features exclusive to AR and VR, such as true‐to‐scale spatial understanding via clinical superimposition, the current preference for segmentation might shift, paving the way for broader adoption of these more advanced technologies.

The analysis of canal detection provided a more nuanced result. Although a statistically significant global difference was found, the effect size was small (*W* = 0.10), and no specific method proved superior in post hoc tests. This suggests that for the fundamental task of identifying canals, all 3D methods are perceived as largely comparable once the initial cognitive hurdle of 2D interpretation is overcome. The trend towards significance for VR over CBCT (*p* = 0.0106) may indicate that the immersive, true‐to‐scale nature of VR offers subtle benefits in spatial understanding, but this advantage was not powerful enough to reach statistical significance in the cohort. This finding is critical, as it implies that the primary benefit of immersive technology may not be in finding more anatomy, but in understanding the relationships of that anatomy with greater confidence and less cognitive effort.

Several limitations of this study must be acknowledged. First, the sample size of 30 participants, while sufficient for the statistical methods employed, is relatively modest. Furthermore, the evaluation was restricted to two specific clinical cases representing common yet challenging anatomical variations (distal bifurcation in a mandibular molar, MB2 confluent with MB1 in a maxillary molar). These cases represent only a fraction of the anatomical complexity encountered in endodontics. This may limit the generalizability of the findings. Perceptions may differ for other challenging scenarios such as C‐shaped canals, dens invaginatus, external/internal resorption, or periapical pathology. Future studies should include a broader range of case complexities and pathological conditions to improve clinical validity and generalizability. The perceptions of the technologies could differ when applied to other challenging scenarios. Second, the study was conducted in a controlled, hypothetical environment. Clinicians were not under the time pressures of a real‐world practice, which could influence usability ratings. The absence of haptic feedback in the VR/AR modalities also represents a significant deviation from a true clinical experience. Third, the ‘novelty effect’ of using immersive technology for the first time may have influenced participant engagement and ratings. Finally, this study focused on perception and usability, not on diagnostic accuracy or impact on treatment outcomes, which remain critical unanswered questions. Furthermore, because participation was voluntary, the sample may overrepresent clinicians who are already enthusiastic about emerging digital technologies. This self‐selection bias limits external validity; those less comfortable with AR/VR or with limited access to such tools might experience different learning curves and outcomes. Future studies should employ random sampling across multiple institutions to mitigate this effect. The accuracy of Diagnocat segmentation was not independently validated for the specific cases used in this study. While the AI driven segmentations have demonstrated acceptable accuracy in published validation studies (Hernandez et al. 2025; Sinard et al. 2025; Fontenele et al. 2023), segmentation performance can vary with image quality, anatomical complexity, and pathological conditions. The two cases selected did not include complex pathologies such as resorption or calcification that may challenge AI algorithms.

A critical methodological consideration is that the comparison inherently conflates two factors: the segmentation process and the visualisation modality. Because all advanced methods utilised pre‐segmented 3D models while conventional CBCT relied on unsegmented slice‐by‐slice analysis, the observed differences cannot be attributed solely to the visualisation format. This design reflects the clinical reality where segmentation is a prerequisite for any 3D or immersive visualisation. Importantly, clinicians' perceptions of the complete diagnostic workflow were evaluated rather than isolated technological components. Future studies incorporating control conditions, such as unsegmented volumetric rendering in OsiriX or segmented models displayed as 2D maximum intensity projections, would be necessary to isolate modality‐specific effects. Such designs would help determine whether the observed advantages stem primarily from the segmentation itself, the 3D visualisation, or their combination. Device‐specific factors including display resolution, field of view, refresh rate, and input modalities differed across platforms and may have influenced usability and information extraction ratings. While this reflects the practical reality of deploying these technologies clinically, it prevents definitive attribution of effects to visualisation modality versus hardware characteristics. Future studies using matched display parameters across conditions could help isolate modality‐specific contributions. The CBCT baseline condition utilised standard MPR viewing in OsiriX Lite without advanced visualisation features such as curved planar reformatting or volumetric rendering. While this reflects typical clinical practice, commercial CBCT software packages increasingly incorporate sophisticated 3D visualisation tools that may narrow the gap with dedicated segmentation platforms. Future comparative studies should evaluate these advanced CBCT features alongside immersive technologies. This study assessed perceived cognitive burden and usability rather than objective cognitive workload.

Our findings are specific to the hardware configurations employed. As hardware evolves rapidly, findings should be interpreted within the context of the specific device generation tested.

While our findings suggest reduced perceived cognitive burden with advanced visualisation methods, this was not directly measured using validated instruments such as the NASA Task Load Index (NASA‐TLX) or physiological proxies (e.g., pupillometry, electroencephalography). Future studies should incorporate such measures to objectively quantify cognitive load differences between visualisation modalities. The fixed presentation order, with CBCT always evaluated first, may have introduced learning effects that systematically disadvantaged the baseline condition. Participants may have developed mental models of the anatomy during initial CBCT evaluation that facilitated subsequent 3D interpretation. However, this fixed order was chosen to reflect the clinical workflow where CBCT interpretation necessarily precedes any advanced visualisation (which requires prior data acquisition). Conversely, fatigue effects from the overall session duration could have negatively impacted ratings for later modalities. While the three advanced modalities were fully counterbalanced among themselves, the CBCT‐first design represents a methodological trade‐off between ecological validity and experimental control.

Critically, this study evaluated clinicians' perceptions and subjective ease of information extraction, not objective diagnostic accuracy. A clinician may report high confidence in identifying root canals using a particular visualisation method while still missing anatomical details due to segmentation errors, display limitations, or cognitive biases. The gap between perceived and actual performance, which is sometimes termed the ‘confidence‐accuracy paradox’, is well‐documented in medical decision‐making literature (Gottlieb et al. 2022; Hautz et al. 2019; Simard‐Sauriol et al. 2025). Validating perceptions against a gold standard (e.g., micro‐CT imaging, histological sectioning) represents the essential next step in this research program. Until such validation is performed, our findings should be interpreted as evidence of user preference and perceived usability rather than diagnostic superiority. The ‘added value’ demonstrated here lies in workflow acceptability and clinician engagement, a prerequisites for technology adoption, but clinical efficacy remains to be established.

Future research should aim to validate these findings with larger, more diverse cohorts and investigate the impact of these technologies on measurable clinical outcomes, such as diagnostic accuracy, treatment time, and patient‐reported outcomes. Longitudinal studies are needed to determine if the perceived benefits of VR and AR are sustained after the initial novelty wears off.

For the VirtualEndo software, future development could focus on several key areas:

1. Direct AI Integration: Integrating AI‐driven segmentation directly into the add‐on to create a seamless ‘CBCT‐to‐VR’ pipeline.
2. Cloud‐Based Processing: Enabling users to upload CBCT scans to a web portal for automated conversion, removing the need for local software installation.
3. Enhanced Interactivity: Adding features for virtual measurements, treatment simulation (e.g., virtual file placement), and collaborative multi‐user sessions for consultations or teaching.

In conclusion, this study provides strong evidence that modern visualisation technologies like Segmentation, VR, and AR are perceived by clinicians as significantly superior to conventional 2D‐slice analysis of CBCT data. While pragmatic concerns may currently favour screen‐based 3D segmentation, the door is clearly open for more immersive technologies to redefine the digital standard of care in endodontics, if workflow barriers continue to be addressed.

## Author Contributions

**Marcel Reymus:** conceptualization, methodology, software, investigation, writing, supervision. **Nils Werner:** conceptualization, methodology, analysis, data curation, writing. **Falk Schwendicke:** writing, supervision. **Ralf Krug:** investigation, validation, writing.

## Conflicts of Interest

The authors declare no conflicts of interest.

## Supporting information


**Supporting Information: 1.** Survey questionnaire.

## Data Availability

The data that support the findings of this study are available from the corresponding author upon reasonable request.
