# Supplementary material for: Practitioners' Perceptions of Augmented and Virtual Reality in Endodontic Treatment Planning: A Pilot Study
Source: Int Endod J. 2026 Mar 15;59(8):1710–20. doi: 10.1111/iej.70143 (PMC13373033; doi:10.1111/iej.70143)
Supplement: Supplementary file 1 — Supporting Information: 1. Survey questionnaire. [file IEJ-59-1710-s001.docx]

Supplemental 1. Survey questionnaire

Part 1: Questionnaire for Clinical Cases 1 and 2

1./5. How well does the CBCT enable you to identify the number and course of the root canals of the tooth?

- [ ] Very good

- [ ] Good

- [ ] Moderate

- [ ] Inadequate

2./6. How well does the segmented view enable you to identify the number and course of the root canals of the tooth?

- [ ] Very good

- [ ] Good

- [ ] Moderate

- [ ] Inadequate

3./7. How well does the VR representation enable you to identify the number and course of the root canals of the tooth?

- [ ] Very good

- [ ] Good

- [ ] Moderate

- [ ] Inadequate

4./8. How well does the AR representation enable you to identify the number and course of the root canals of the tooth?

- [ ] Very good

- [ ] Good

- [ ] Moderate

- [ ] Inadequate

Part 2: Usability of different technologies

9. How do you rate the usability of the CBCT data when planning an endodontic treatment?

- [ ] Very easy

- [ ] Easy

- [ ] Moderate

- [ ] Difficult

10. H**ow do you rate the usability of the segmented view for planning endodontic treatment?**

- [ ] Very easy

- [ ] Easy

- [ ] Moderate

- [ ] Inadequate

11. **How do you rate the user experience of the** VR visualization **for planning endodontic treatment?**

- [ ] Very easy

- [ ] Easy

- [ ] Moderate

- [ ] Difficult

12. **How do you rate the usability of the AR overlay for endodontic treatment planning?**

- [ ] Very easy

- [ ] Easy

- [ ] Moderate

- [ ] Difficult

13. **How easy is it to extract the following information from CBCT (cone-beam CT) data**?

a) Number of canals: [ ] Very Easy [ ] Easy [ ] Moderate [ ] Difficult

b) Course of Canals: [ ] Very Easy [ ] Easy [ ] Moderate [ ] Difficult

14. **How easy is it to extract the same information from the segmented view?**

a) Number of canals: [ ] Very Easy [ ] Easy [ ] Moderate [ ] Difficult

b) Course of Canals: [ ] Very Easy [ ] Easy [ ] Moderate [ ] Difficult

15. **How easy is it to identify and extract the same information from the VR visualization?**

a) Number of canals: [ ] Very Easy [ ] Easy [ ] Moderate [ ] Difficult

b) Course of Canals: [ ] Very Easy [ ] Easy [ ] Moderate [ ] Difficult

16. **How easy is it to access and interpret the same information from the AR overlay?**

a) Number of canals: [ ] Very Easy [ ] Easy [ ] Moderate [ ] Difficult

b) Course of Canals: [ ] Very Easy [ ] Easy [ ] Moderate [ ] Difficult

Part 3: Clinical Relevance

17. Which method provides, in your opinion, the more clinically relevant information for endodontic treatment?

- [ ] CBCT

- [ ] Segmentation

- [ ] VR

- [ ] AR

- [ ] all the same
